# Neural Correlates of Spatial Navigation Changes in Mild Cognitive Impairment and Alzheimer’s Disease

**DOI:** 10.3389/fnbeh.2014.00089

**Published:** 2014-03-17

**Authors:** Kamil Vlček, Jan Laczó

**Affiliations:** ^1^Department of Neurophysiology of Memory, Institute of Physiology, Academy of Sciences of the Czech Republic, Prague, Czech Republic; ^2^International Clinical Research Center, St. Anne’s University Hospital Brno, Brno, Czech Republic; ^3^Department of Neurology, 2nd Faculty of Medicine, Charles University in Prague and Motol University Hospital, Prague, Czech Republic

**Keywords:** Alzheimer’s disease, mild cognitive impairment, spatial navigation, spatial disorientation, brain changes

## Abstract

Although the memory impairment is a hallmark of Alzheimer’s disease (AD), AD has also been characterized by spatial disorientation, which is present from its early stages. Spatial disorientation in AD manifests itself in getting lost in familiar and unfamiliar places and have been characterized more specifically using spatial navigation tests in both real space and virtual environments as an impairment in multiple spatial abilities, including allocentric and egocentric navigation strategies, visuo-spatial perception, or selection of relevant information for successful navigation. Patients suffering mild cognitive impairment (MCI), who are at a high risk of development of dementia, show impairment in a subset of these abilities, mainly connected with allocentric and egocentric processing. While spatial disorientation in typical AD patients probably reflects neurodegenerative changes in medial and posterior temporal, parietal, and frontal lobes, and retrosplenial cortex, the impairment of spatial navigation in MCI seem to be connected mainly with the medial temporal and also parietal brain changes. In this review, we will summarize the signs of brain disease in most MCI and AD patients showing in various tasks of spatial memory and navigation.

## Introduction

Spatial disorientation is one of the early manifestations of Alzheimer’s disease (AD), besides the clinically mostly used memory impairment. The research in spatial deficits in this neurodegenerative disease has grown rapidly in last years and decline in spatial navigation abilities may become another diagnostic mark for AD in the near future. Spatial navigation is however not a unitary function. This ability to determine and maintain a route from one place to another (Gallistel, 1990) utilizes multiple spatial strategies recruiting distinct brain regions.

This review aims to describe spatial disorientation in AD and mild cognitive impairment (MCI) as a multi-factorial deficit connected with changes in several brain regions. Various described manifestations of these changes in spatial cognitive tasks are the focus of this article. Selected for the review were only studies describing impairment in AD or MCI patients in real or virtual space, in spatial navigation, or associated abilities like perspective taking or object location memory.

## Current View on Spatial Deficits in AD and MCI

A number of studies focusing on visuo-spatial deficits in AD and MCI appeared during last two decades. The main published view on these deficits is broadly dual: one series of studies documented visuo-perceptual nature of the disorientation, its association with optic flow perception, and visuo-spatial attention (e.g., Tetewsky and Duffy, 1999; Cherrier et al., 2001; Mapstone et al., 2003; Kavcic et al., 2006). Another series of investigations stressed, however, the cognitive mapping deficits in these patients, specifically in using the allocentric navigation (Kalova et al., 2005; Hort et al., 2007; Weniger et al., 2011; Nedelska et al., 2012). According to some other reports, the spatial disorientation in AD and MCI patients seem to be associated with both medial temporal and parietal lobe function (Henderson et al., 1989; deIpolyi et al., 2007). Several reviews on this theme published recently support either the latter allocentric view (Iachini et al., 2009) or combination of both cognitive mapping and visuo-perceptual factors (Vlcek, 2011; Gazova et al., 2012) or suggest a multifocal theory of disease developing from temporal to parietal and lateral to frontal brain and midbrain and associated cognitive deficits (Lithfous et al., 2013). One other review proposes the translation between egocentric and allocentric frames, supported by retrosplenial cortex (RSC), being the basis of spatial disorientation deficits in MCI and AD (Serino and Riva, 2013).

## Brain Changes in MCI and AD

The anterior medial temporal lobe structures are the first affected by AD pathology. Histopathological changes initially occur in the entorhinal cortex and the hippocampus, further spread throughout parahippocampal gyrus to the temporal pole and inferior and middle temporal gyri in MCI and preclinical AD, and subsequently spread throughout the temporal, parietal, and frontal neocortex by the time of dementia due to AD (Braak and Braak, 1995; Petersen et al., 2006). In agreement with this neuropathological staging, the highest rate of atrophy in the MCI and initial stages of AD has been found in the entorhinal and perirhinal cortices and the hippocampus (Pennanen et al., 2004; Schmidt-Wilcke et al., 2009; Risacher et al., 2010), which also showed accelerated volume loss over the time (Schuff et al., 2012) and hypometabolism (Karow et al., 2010). The anteromedial temporal atrophy was described even in cognitively normal individuals later converting to MCI (Smith et al., 2007). The posterior part of the gyrus, the parahippocampal cortex, is affected later in the course of AD (Karow et al., 2010; Spulber et al., 2012), followed by atrophy of the fusiform gyrus (McDonald et al., 2009).

A number of neuroimaging studies have also shown structural and metabolic changes in the parietal lobe, early in the course of AD. Cortical atrophy (Fennema-Notestine et al., 2009) in the precuneus and the inferior parietal lobule were reduced in the early MCI stages (McDonald et al., 2009) and volume reduction of these areas is the most consistent finding among the MCI to AD converters (Karas et al., 2008; Whitwell et al., 2008) and was described even in normal individuals later converting to MCI and AD (Smith et al., 2007; Jacobs et al., 2011). Hypometabolism was also found in superior parietal lobules (Li et al., 2008; Nobili et al., 2008) and even more in the inferior parietal lobule (Nobili et al., 2009) in MCI patients, especially those converting later to AD (Drzezga et al., 2003; Hirao et al., 2005).

Within the cingulate gyrus, the posterior cingulate and RSC are affected early in the course of AD. Atrophy of these areas was demonstrated in mild AD patients (Scahill et al., 2002) and patients with early stages of MCI (Chetelat et al., 2002; Fennema-Notestine et al., 2009), especially in those later progressing to AD (Hamalainen et al., 2007; Whitwell et al., 2008; Julkunen et al., 2009; Pengas et al., 2010a). Severe posterior cingulate cortex hypometabolism is a feature of incipient AD (Nestor et al., 2003a,b) and is present already in the MCI patients (Huang et al., 2002; Ishiwata et al., 2006; Johnson et al., 2007; Pappata et al., 2010).

Neuropathological changes occur in the frontal cortex later in the course of AD (Braak and Braak, 1995; Petersen et al., 2006). Frontal lobe atrophy and hypometabolism is not present earlier than at the later stages of MCI and mainly in the prefrontal cortex (Fennema-Notestine et al., 2009; Langbaum et al., 2009; McDonald et al., 2009) but is more pronounced in those MCI patients who later converted to AD (Drzezga et al., 2003; Whitwell et al., 2008).

Although the described the brain changes prevails in AD and MCI patients, in a significant portion of AD patients the underlying neuropathological process follows alternative distribution, representing at least two other clinicopathological subtypes of AD and contrasting with the typical AD (Murray et al., 2011). In the hippocampal sparing AD subtype, found in 11% of patients, the neuronal degeneration results in lower gray matter volumes of lateral parietal, lateral temporal, and lateral frontal cortex, compared to typical AD (Whitwell et al., 2012). In the limbic-predominant AD subtype, found in 14–19% patients, the areas affected more than in the typical AD are the hippocampus and amygdala, with lower gray matter volumes. These differences are associated with distinct cognitive profiles in memory and other cognitive domains and necessarily also in spatial navigation, as well as with the different course of cognitive changes over time. However, the AD subtypes have not been considered in the studies reviewed below and the described deficits in spatial memory applies probably only to the predominating typical AD.

## Spatial Navigation Deficits in MCI and AD

### Allocentric navigation, object location memory, and scene processing

Allocentric memory enables us to locate a goal in relation to surrounding objects and global landmarks, a function localized to medial temporal lobe, and hippocampus specifically (O’Keefe and Nadel, 1978; Maguire et al., 1998; Astur et al., 2002; Feigenbaum and Morris, 2004; Parslow et al., 2004). Hippocampal role in spatial deficits in AD and MCI have been documented by a series of allocentric navigation studies: AD patients were impaired in a real space analog of Morris water maze, termed blue velvet arena (BVA): only in allocentric trials when the cues on the wall could be used for orientation, but not in trials without cues, when only the start position could be used (Kalova et al., 2005). In the same apparatus, a more strictly defined AD group had problems navigating using both start position and cues on the walls, but an amnestic MCI single-domain group was impaired only in the allocentric trials (Hort et al., 2007), suggesting specific hippocampal impairment. This was supported later in a follow-up study where hippocampal amnestic MCI patients showed no learning in the allocentric trials (Laczo et al., 2009). Virtual analogy was also used in a recent study (Hort et al., 2014), where BVA was termed Urania. Amnestic MCI were also impaired in another allocentric navigation test to find shortest way to hidden targets in a virtual park (Weniger et al., 2011). A connection of allocentric navigation to hippocampal function was supported also in a study correlating real space Morris water maze analogy navigation successfulness with right hippocampal volume (Nedelska et al., 2012).

Successfulness in other spatial tasks is probably also connected to hippocampal function. Memory for temporal sequence of three body turns in a Starmaze was documented to activate left hippocampus (Igloi et al., 2010) and later shown to distinguish well between mild AD patients and controls (Bellassen et al., 2012). Memory for location of objects in space was several time consistently shown to be dependent on hippocampal function (Milner et al., 1997; Kessels et al., 2004; Stepankova et al., 2004) and reported to be deficient in patients suffering AD (Bucks and Willison, 1997; Brandt et al., 2005) and also in MCI patients, although to a lesser degree than in AD (Kessels et al., 2010). In contrast, memory for several positions without objects seem to be preserved even in mild AD (Adelstein et al., 1992; Kalova et al., 2005).

Processing of viewpoint independent spatial representation of a scene during scene matching seem also to be associated with hippocampal function after very short delays and with parahippocampal cortex function even in the presence of the sample scene (Hartley et al., 2007). In the same test, groups of six AD patients and seven amnestic MCI patients were impaired after short delays but not in direct scene matching (Bird et al., 2010), while a larger group of AD patients was impaired even in matching of simultaneously visible scenes (Pengas et al., 2010b). In a similar test, scene discrimination across different views was worsened in a selective hippocampal damage group (Lee et al., 2005) and also in a group of mild AD patients (Lee et al., 2006).

### Reference frame translation

Retrosplenial cortex, a part of the posterior cingulate cortex, is strongly involved in spatial processing, specifically in translation between egocentric and allocentric representations (Byrne et al., 2007). Its damage shows as heading disorientation, an inability to derive directional information from scenes or to estimate spatial relationship between two locations (Aguirre and D’Esposito, 1999). Impairment of head orientation was also documented in AD but not MCI patients, reaching lower score in head orientation test, requiring to indicate directions after a test of navigation within a virtual city (Pengas et al., 2010b). Navigation score in this test correlated with gray matter density and glucose metabolism in RSC, but also hippocampus (Pengas et al., 2012). In another study on virtual as well as real navigation in a hospital lobby, AD patients, but again not MCI patients, were impaired in a test of self-orientation, requiring to indicate directions to scenes from the route (Cushman et al., 2008). The AD patients were also impaired in navigation in a virtual-reality maze using its map, which required translation of allocentric representation in the map to the egocentric direction in the maze (Morganti et al., 2013). Location of scenes on a map could possibly be also regarded as a behavioral measure of RSC function, requiring egocentric to allocentric translation. This ability was impaired in MCI patients in two studies focused on route learning and follow-up set of spatial tests (deIpolyi et al., 2007; Cushman et al., 2008).

### Egocentric navigation

Egocentric memory enables us to remember positions in space in relation to one’s own position and heading in space. The brain localization of the navigational strategy using egocentric reference frame seems to be diverse and possibly reflects multiplicity of strategies concealed under the usage of a single egocentric reference frame. The superior and inferior parietal lobe structures have been activated during various egocentric tasks in Morris water maze analogy (Parslow et al., 2004), virtual city (Maguire et al., 1998; Wolbers et al., 2004) as well as in landmark-free environment (Wolbers et al., 2008). The activity in caudated nucleus was associated with a response strategy in a virtual analogy of a radial maze (Iaria et al., 2003) and following a well-learned route in a virtual city (Hartley et al., 2003).

Egocentric navigation was documented to be impaired in AD and also MCI patients in two types of experiments. In a both real space and computer analogy of Morris water maze, amnestic MCI patients with associated non-memory impairment scored similarly to AD patients in finding of hidden goal position using only their starting position (Hort et al., 2007). In landmark-free virtual-reality maze, requiring the subjects to use only the sequence of egocentric turns during navigation, the amnestic MCI subjects were unable to learn a route to a hidden reward (Weniger et al., 2011). In addition, the number of errors in this maze correlated negatively with precuneus volume, supporting the assumption of egocentric strategy use.

### Visual perception

A wealth of reports document the role of visual perception functions in navigational impairment: either perception of optic flow, visuo-spatial attention, or visual perceptual analysis. The optic flow perception is supported by the visual area V5/MT at the junction of occipital, temporal, and parietal lobes (Morrone et al., 2000). Connection of optic flow perception thresholds with navigation impairment in AD patients was described in studies using route learning in a hospital lobby (Tetewsky and Duffy, 1999) or indirectly using left–right orientation in table-top Money Road Map test (MRMT) and sustained driving in On-the-Road Driving test (O’Brien et al., 2001). Similar correlation between MRMT scores and optic flow perception was found also in MCI patients (Mapstone et al., 2003). Even better prediction of the navigation score in a hospital lobby was found in a combined regression model containing contrast sensitivity score and amplitude of the visual ERP N200 responses (Kavcic et al., 2006). This association of navigation score to visual perception could however be confined only to men patients (Cushman and Duffy, 2007), while in women patients the navigation score seem to be better predicted by verbal fluency and figural memory.

The perceptual nature of disorientation could be inferred also from other studies on AD patients. These patients were impaired in recognition of incidental landmarks not mentioned during the walk in a hospital lobby in contrast to correct recognition of the mentioned landmarks (Cherrier et al., 2001). In another study, also using route learning in a hospital lobby, the impairment of AD patients was predicted by MRMT and Line orientation test but not by mostly low memory scores (Monacelli et al., 2003). The impairment of AD patients in all angle categories of turns in MRMT, in combination with their normal left–right discrimination, have also been explained by visual perceptual deficits (Rainville et al., 2002). Similarly, visuoconstructive test scores together with results from a memory test predicted spatial disorientation sub-score from Memory and Behavior Problems Checklist questionnaire (Henderson et al., 1989), supporting the dual roots of AD disorientation.

### Planning and problem solving

Deficits in frontal problem solving functions were documented in a unique experiment (Passini et al., 1995), requiring AD subjects to guide the experimenter to the dental clinic in an unknown hospital and to express verbally everything that went through their mind. To minimize the effect of memory and attentional deficit, the subjects were repeatedly reminded about their task. Their behavior was seemingly more driven by external stimuli than by the goal of the way-finding task, suggesting difficulties to distinguish relevant from irrelevant information and to structure their decision plan.

### Landmark recognition

Individual recognition of landmarks, salient features of environment useful for navigation, is an isolated cognitive ability, impaired in landmark agnosia (Aguirre and D’Esposito, 1999) and dependent on the function of the anterior end of the right lingual gyrus (Aguirre et al., 1998; Mendez and Cherrier, 2003). Three real-world navigation studies report different successfulness in AD and MCI patients: in a series of tests after a walk in a hospital lobby, MCI and mild AD patients were similar to controls in landmarks recognition (deIpolyi et al., 2007), but were impaired in a more recent study (Benke et al., 2013). The relationship of this impairment to visuo-spatial attention are suggesting the results of an earlier study, where recognition of landmarks mentioned by experimenter during the walk was least impaired in AD patients, in contrast to their large impairment in recognition of landmarks not mentioned during the walk (Cherrier et al., 2001).

In contrast, in a free recall of landmarks along a route, the AD patients were found to be impaired (Monacelli et al., 2003) and this measure even distinguished reliably between MCI and healthy old subjects (Cushman et al., 2008).

## Conclusion

Findings of this survey are mainly consistent with the described brain changes during spreading of the disease and suggest its propagation from the anterior medial temporal lobe to posterior temporal and parietal areas in MCI and to frontal and parieto-occipital areas later in AD patients. MCI patients seem to be impaired first in the allocentric navigation and later in the multi-domain stage also in egocentric navigation. Consistently with this double impairment, suggesting their medial temporal as well as posterior parietal damage, they have been found to be impaired also in route learning in both real and virtual environments. Short-term scene memory, visuo-spatial attention, and optic flow perception may also affect their navigational successfulness.

The broad cognitive impairment of even mild AD patients interferes also with other abilities essential for successful navigation, as optic flow perception, reference frame translation, scene matching, spatial planning, visual perceptual analyses, and possibly landmark recognition. Their navigation difficulties seem therefore to be connected with their more wide spread brain damage in other areas of parietal lobes and temporal cortex, RSC, as well as frontal lobes.

## Author Contributions

Both Kamil Vlček and Jan Laczó wrote and discussed the manuscript, contributed to the final version of the paper and have approved it.

## Conflict of Interest Statement

Dr. Jan Laczó has consulted for Pfizer and holds shares of Polyhymnia-TS. Authors declare that the research was conducted in the absence of any other commercial or financial relationships that could be construed as a potential conflict of interest.
